# Dorsal penile nerve block alleviates pain in men undergoing rigid cystoscopy: A single‐center, randomized, double‐blind, and placebo‐controlled trial

**DOI:** 10.1002/bco2.76

**Published:** 2021-02-05

**Authors:** Yan Qiu, Xinhao Liu, Wuran Wei, Guizhi Du

**Affiliations:** ^1^ Department of Anesthesiology West China Hospital of Sichuan University Chengdu China; ^2^ Department of Urinary Surgery West China Hospital of Sichuan University Chengdu China

**Keywords:** analgesia, dorsal penile nerve block, local anesthesia, men, rigid cystoscopy

## Abstract

**Objective:**

To assess the effectiveness and safety of dorsal penile nerve block (DPNB) compared with tetracaine gel in analgesia for men undergoing rigid cystoscopy.

**Patients and methods:**

This study was conducted as a prospective, randomized, double‐blind, placebo‐controlled, and single‐center trial. Men undergoing diagnostic rigid cystoscopy were randomly allocated into one of three groups (n = 86 each): (1) tetracaine gel group (DPNB with saline), (2) DPNB group (DPNB with ropivacaine + plain lubricant), and (3) combination group (DPNB with ropivacaine + tetracaine gel). The primary outcome was visual analog scale (VAS) for pain at cystoscopic inspection of the external sphincter.

**Results:**

VAS (median [interquartile]) at inspection of the external sphincter was significantly lower in both DPNB and combination groups than that in tetracaine gel group (4 [3–6] and 4 [3–5] vs 6 [5–7], *P* < .0001), with a 33% reduction of median VAS. Overall pain level during procedure was significantly alleviated in the DPNB and combination groups with more stabilized HR and MAP when compared with tetracaine gel group. No adverse events were observed during DPNB including penile hematoma, erection, local anesthetic toxicosis or incompletion of cystoscopy.

**Conclusion:**

Our study suggests analgesia provided by DPNB with or without tetracaine gel instilled is superior to that provided by tetracaine gel alone in men undergoing diagnostic rigid cystoscopy.

**Clinical Trials Registration:**

ClinicalTrials.gov registration number: NCT02502487.

## 1 INTRODUCTION

Cystoscopy is a kind of invasive manipulation often used by urologists for diagnosis and treatment of bladder cancer and other lower urinary tract diseases.^1^ Pain and discomfort is common in men undergoing cystoscopy even under general anesthesia or with topical administration of local anesthetic.^2^, ^3^, ^4^ Under general anesthesia, 30% patients complain of pain within 1 week after rigid cystoscopy.^3^ In outpatient settings, even with application of a lubricant containing 2% lidocaine, about 76% men suffer from mild to severe pain during rigid cystoscopy, and approximately 27% patients could still feel mild to moderate pain 7 days after the procedure.^4^ As for flexible cystoscopy, most patients also feel pain during the procedure with topical instillation of lidocaine gel.^5^ While flexible cystoscopy is widely used in developed countries, rigid cystoscopy is still mostly used in developing countries because it is cost‐effective and easy to use and provides better optical area.^6^ Thus, an effective analgesia regimen seems to be urgent for men undergoing rigid cystoscopy.

Since the urethra is longer and narrower in men, cystoscopy usually tends to be more painful for them.^7^ The procedure is most painful for men when the cystoscope passes through the membranous urethra.^8^ Song et al.^9^ did autopsy on males and found the dorsal nerve of the penis (DNP), the terminal branch of the pudendal nerve, innervates the membranous urethra. In addition, urethral mucosa is innervated by branches of DNP.^10^

Currently dorsal penile nerve block (DPNB) is usually used for circumcision in children and it has been shown to provide effective analgesia for penile surgeries since it was first reported by Kirya and Werthmann in 1978.^11^ To date, whether DPNB could be applied in cystoscopy for analgesia has not been addressed. Since DNP branches innervate membranous urethra and urethra mucosa, we hypothesized that DPNB could reduce overall pain level in men during rigid cystoscopy.

## 2 PATIENTS AND METHODS

### 2.1 Trial authorization

This trial received Biomedical Ethics Committee approval at West China Hospital of Sichuan University (Ref: 20150611) and was registered at Clinicaltrials.gov (NCT02502487). This study was conducted at West China Hospital of Sichuan University.

### 2.2 Subjects

We enrolled adult male patients aged 20 to 75 yr undergoing diagnostic rigid cystoscopy, with American Society of Anesthesiologists (ASA) physical classification I to II, without history of urethral or prostatic surgery, respiration or circulation disorders, or chronic pain. All participating subjects provided written informed consent to participate in this single‐center, randomized, double‐blind, placebo‐controlled clinical trial. Patients were excluded in case of allergy to local anesthetics; coagulation disorder or usage of antiplatelet drugs; infection at the site of DPNB puncture point; severe urethral stenosis.

### 2.3 Randomization and blinding

All included subjects were randomly enrolled and allocated into one of three groups with a 1:1:1 ratio by sealed, opaque assignment envelope. The randomization number in the envelope was generated with a computerized SPSS software package (version 18; SPSS Inc., Chicago, IL). The three groups are as follows: tetracaine gel (DPNB with saline + tetracaine gel), DPNB (DPNB with ropivacaine + plain lubricant), or combination group (DPNB with ropivacaine + tetracaine gel). On the day of cystoscopy, the nurse who opened the sealed envelopes, prepared the syringe containing either 0.3 mL/kg of 0.33% ropivacaine (AstraZeneca Pharmaceutical, Inc., London, United Kingdom) or 0.3 mL/kg of saline for DPNB, and 10 mL of 1% tetracaine gel (Xi'an Lijun Pharmaceutical Co., Ltd, Xi'an, China) or 10 mL of liquid glycerin (YunJia Medical Technology Co., Ltd., Harbin, China) for instillation. All patients received topical intraurethral anesthesia after DPNB for the procedure. All trial participants, attending anesthesiologist and urologist as well as investigators were not aware of randomization. Allocation concealment was not exposed until the final data analysis report was completed.

### 2.4 Pre‐cystoscopy procedures

Intravenous access was established and pulse oximetry, noninvasive brachial blood pressure, and electrocardiograph were applied upon patient arrival to the block room. Regardless of group allocation, block sties were similarly sterilized in all patients.

DPNB Group. After skin preparation and palpation of the arch of the lower border of symphysis pubis, the base of the penis was gently pulled down, and DPNB was performed using a 22‐G needle inserted on either side of the midline just distal to the inferior ramus of the pubic bone and advanced slowly toward the center of the penile shaft until loss of resistance was felt as penetrating Scarpa's fascia, where 0.3 mL/kg of 0.33% ropivacaine was deposited equally in each side. All blocks were performed by one experienced attending anesthesiologist. Five minutes after DPNB, 10 mL of liquid glycerin were instilled in the DPNB group, with a dwell time of 10 min before rigid cystoscopy.

Combination Group of DPNB and Tetracaine Gel. After skin preparation, DPNB with 0.33% ropivacaine (0.3 mL/kg) was performed as in the DPNB group. Five minutes after DPNB, 10 mL of 1% tetracaine gel was instilled in the combination group, with a dwell time of 10 min before rigid cystoscopy.

Control Group of Tetracaine Gel. After skin preparation, DPNB is performed with saline (0.3 mL/kg) in the tetracaine gel group. Five minutes after DPNB, 10 mL of 1% tetracaine gel was instilled in the tetracaine group, with a dwell time of 10 min before rigid cystoscopy.

### 2.5 Outcome measures

Each patient characteristics including age, weight, height, body mass index, duration of rigid cystoscopy, first‐time or repeat cystoscopy, visual analog scale (VAS) scores, vital signs and the incidence of adverse events during rigid cystoscopy were collected in a designed data form by an investigator.

The intensity of pain measured by a VAS at cystoscopic inspection of the external sphincter was designated as the primary outcome. VAS is an internationally recognized pain scale with 11 points (0 = no pain, 10 = maximal pain). Pain can be rated as the following categories: no pain (0 points), mild pain (1–3 points), moderate pain (4–6 points), and severe pain (7–10 points). Participants were well educated for VAS once enrolled and asked to rate their pain levels during the study.

Secondary outcomes included: VAS scores, assessed prior to lubricant administration, at inspection of the penile and bulbar urethra, at inspection of the prostate and the bladder, and at withdrawal of the cystoscope; vital signs (heart rate, blood pressure, breath rate and pulse oxygen saturation) that consider the discomfort and pain during the procedure and recorded during the study; the incidence of adverse events (penile hematoma, penile erection, autonomic movement, local anesthetic toxicosis, involuntary movements due to pain and incompletion of cystoscopy) recorded from the beginning of DPNB to the end of cystoscopy for evaluation of the safety of DPNB in rigid cystoscopy.

### 2.6 Study population size

To detect a minimal 20% difference on the VAS at cystoscopic inspection of the external sphincter between treatment and control groups with 90% power, an *α* of .05, and a standard deviation (SD) of 2.0, we need 78 patients in each group. Consequently, to take into account patients who could not be evaluated, we aimed to enroll a total of 258 patients for this study. The SD was estimated based on the results of our pilot study since there is no literature regarding VAS score for men when rigid cystoscope passes through the membranous urethra.

### 2.7 Statistical analysis

The SPSS for Windows (version 18; SPSS Inc., Chicago, IL) and SAS (version 9.3; SAS Inst. Inc., Cary, NC) were used to perform the analyses. Data with normal distribution were expressed as mean ± SD and tested by one‐way ANOVA with Dunnett's *post hoc* test as appropriate. Continuous data without normal distribution were expressed as median with interquartile range and analyzed using the MIXED procedure of SAS statistical program for repeated measures followed by Tukey‐Kramer adjustments. The significance level of *P*‐value was set at .05 (two‐tailed).

## 3 RESULTS

**FIGURE 1:**
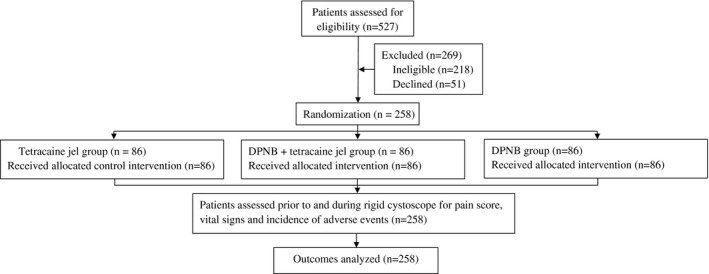
Patient flow chart. DPNB, dorsal penile nerve block

Five hundred and twenty‐seven patients were assessed for eligibility, of which 269 were found either ineligible or declined and 258 were recruited (Figure 1). The characteristics of enrolled patients were similar for the three study groups (Table 1).

**TABLE 1:** Characteristics of the study subjects undergoing rigid cystoscopy

Variables	Tetracaine group (n = 86)	DPNB group (n = 86)	Combination group (n = 86)	*P*‐value
Age (yr)	53.4 ± 11.5	52.1 ± 13.0	53.5 ± 12.0	.71
Weight (kg)	66.8 ± 8.2	68.8 ± 9.6	66.4 ± 8.9	.16
Height (cm)	167.3 ± 5.6	168.7 ± 5.5	167.8 ± 6.4	.26
Body mass index (kg/m^2^)	23.8 ± 2.4	24.2 ± 3.0	3.6 ± 2.7	.35
Duration of cystoscopy (min)	3.5 ± 0.6	3.5 ± 0.5	3.4 ± 0.5	.07
First‐time cystoscopy	60 (30.2%)	58 (32.6%)	61 (29.1%)	.74
Repeat cystoscopy	26 (69.8%)	28 (67.4%)	25 (70.9%)	

Note

Values are presented as mean ± SD or number of subjects (percentage). *P*‐values were derived from one‐way ANOVA for continuous variables or Pearson chi‐square test for categorical variables.

Abbreviation: DPNB, dorsal penile nerve block.

### 3.1 Visual analog scale pain scores

**FIGURE 2:**
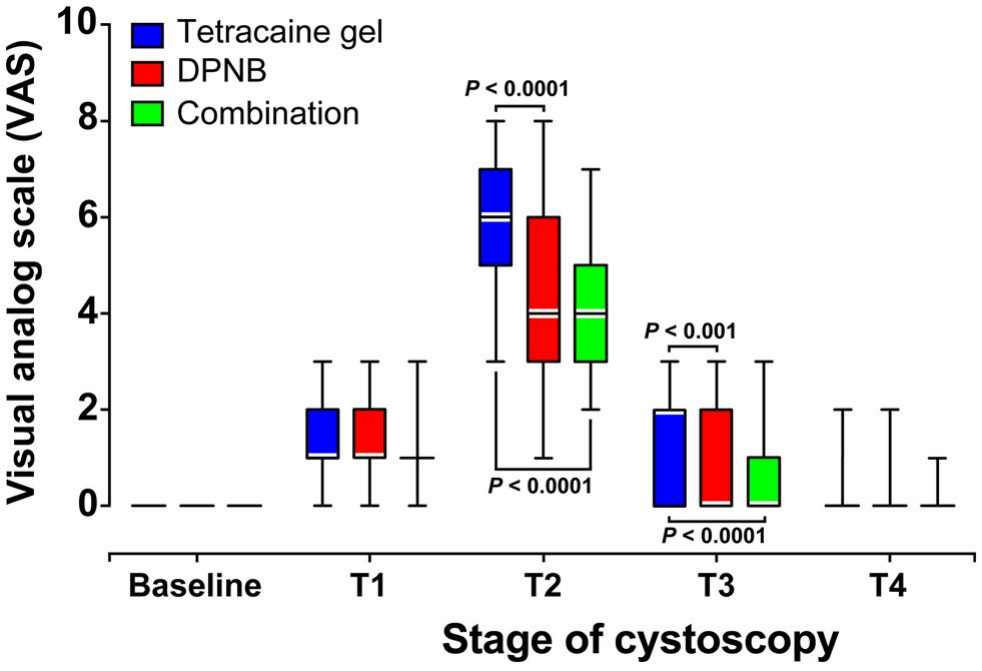
Box plots (median and interquartile range) of pain scores on visual analog scale (VAS) for the three study groups during rigid cystoscopy. During the procedure, VAS scores were significantly reduced in both DPNB and combination groups compared with tetracaine group (*P* < .0001, MIXED procedure with repeated measures for group effect during cystoscopy). Specifically, VAS was significantly lower in DPNB and combination groups at inspections of the external sphincter and of the prostate and bladder, as compared with tetracaine gel group (both *P* < .0001, and *P* < .001 and *P* < .0001, respectively, MIXED procedure for repeated measures followed by Tukey‐Kramer adjustments). DPNB, dorsal penile nerve block; T1, at inspection of penile and bulbar urethra; T2, at inspection of external sphincter; T3, at inspection of prostate and bladder; T4, after withdrawal of cystoscope

Pain severity VAS scores for the three groups during rigid cystoscopy were shown in Figure 2. Overall, MIXED procedure for repeated measures revealed that DPNB and combination groups had less pain compared with tetracaine gel group during rigid cystoscopy (both *P* < .0001). At the primary outcome, pain at cystoscopic inspection of the external sphincter, the VAS pain score was significantly lower in DPNB and combination groups when compared with tetracaine gel group (median [interquartile]: 4 [3–6] and 4 [3–5] vs 6 [5–7], *P* < .0001). Pain score at inspection of prostate and bladder was also lower in both DPNB and combination groups than that in tetracaine group (median [interquartile]: 0 [0–2] and 0 [0–1] vs 2 [0–2], *P* < .001 and *P* < .0001, respectively). Pain severities at inspection of penile and bulbar urethra and after withdrawal of cystoscope were similar among the three groups.

### 3.2 Vital signs

Table 2 shows changes in vital signs for the three groups during rigid cystoscopy. During rigid cystoscopy, DPNB and combination groups had a significantly lower heart rate (HR) and mean arterial pressure (MAP) compared with tetracaine gel group (repeated‐measures ANOVA, both *P* < .001). Specifically, HR and MAP were lower in the DPNB and combination groups at cystoscopic inspection of external sphincter (mean ± SD: 90.2 ± 14.9 and 86.2 ± 10.9 vs 96.7 ± 14.1 beats/min, *P* = .003 and *P* < .001, respectively; 105.8 ± 10.5 and 104.8 ± 9.2 vs 113.7 ± 11.7 mmHg, both *P* < .001) and after withdrawal of cystoscope (mean ± SD: 72.3 ± 10.0 and 71.6 ± 9.2 vs 76.7 ± 10.9 beats/min, *P* = .008 and *P* = .002, respectively; 95.7 ± 8.4 and 95.2 ± 8.6 vs 99.3 ± 9.8 mmHg, *P* = .018 and *P* = .007, respectively), compared with tetracaine gel group. Pulse oxygen saturation (SpO_2_) and respiratory rate (RR) did not differ in the three groups during rigid cystoscopy.

**TABLE 2:** Changes in vital signs during rigid cystoscopy

Variables	Stage of cystoscopy				*P*‐value
Group	Before gel administration	Inspection of penile and bulbar urethra	Inspection of external sphincter	After withdrawal of cystoscope	
*HR (beats/min)*					
Tetracaine gel	75.0 ± 9.7	84.8 ± 11.7	96.7 ± 14.1	76.7 ± 10.9	<.001
DPNB	75.9 ± 9.4	83.8 ± 12.0	90.2 ± 14.9^†^	72.3 ± 10.0^†^	
Combination	73.6 ± 10.2	81.1 ± 10.4	86.2 ± 10.9^‡^	71.6 ± 9.2^†^	
*MAP (mmHg)*					
Tetracaine gel	95.3 ± 10.2	100.0 ± 10.6	113.7 ± 11.7	99.3 ± 9.8	<.001
DPNB	96.6 ± 9.7	99.8 ± 10.1	105.8 ± 10.5^‡^	95.7 ± 8.4*	
Combination	95.1 ± 9.9	98.2 ± 9.0	104.8 ± 9.2^‡^	95.2 ± 8.6^†^	
*SpO_2_ (%)*					
Tetracaine gel	99 (98–99)	99 (98–99)	99 (98–99)	99 (98–99)	.31
DPNB	98 (97–99)	98.5 (98–99)	98 (97–99)	98 (98–99)	
Combination	99 (98–99)	99 (98–99)	99 (98–99)	99 (98–99)	
*RR (breaths/min)*					
Tetracaine gel	19.8 ± 1.1	20.0 ± 1.2	20.1 ± 1.3	19.7 ± 1.2	.19
DPNB	19.6 ± 1.6	19.8 ± 1.5	19.8 ± 1.7	19.4 ± 1.5	
Combination	19.5 ± 1.3	19.6 ± 1.3^†^	19.8 ± 1.5	19.4 ± 1.2	

Notes

Values are presented as mean ± SD or median (interquartile). *P*‐values for group effect during cystoscopy were derived from repeated‐measures ANOVA or MIXED procedure for repeated measures, as appropriate.

Abbreviations: HR, heart rate; MAP, mean arterial pressure; RR, respiratory rate; SpO_2_, pulse oxygen saturation.

* *P* < .05;

^†^ *P* < .01;

^‡^ *P* < .001 vs Tetracaine gel group (multiple comparisons with Tukey‐Kramer adjustments).

### 3.3 Adverse events

**FIGURE 3:**
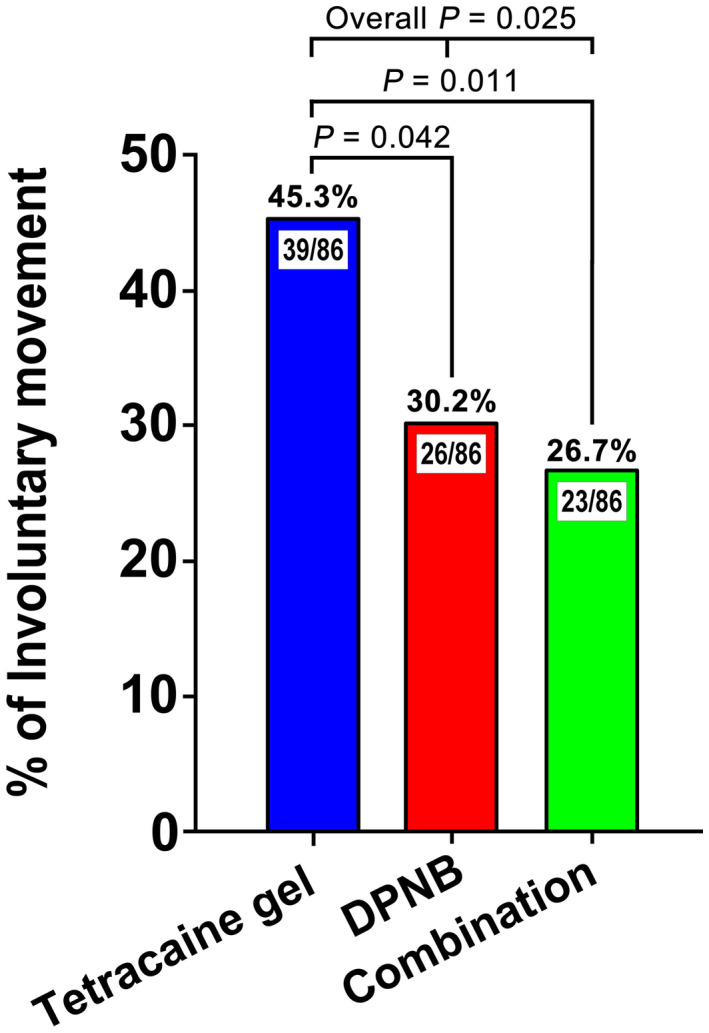
The incidence of involuntary movement due to pain for the three study groups during rigid cystoscopy. In the procedure, the percentage of involuntary movement was lower in both DPNB and combination groups compared with tetracaine group (*P = *.042 and *P = *.011, respectively, Chi‐Square tests using SAS PROC FREQ followed by PROC MULTITEST). DPNB, dorsal penile nerve block

No adverse events were observed in the three groups during the study including penile erection, hematoma, toxicosis and incompletion of cystoscopy. Chi‐Square tests using SAS PROC FREQ followed by PROC MULTITEST revealed that incidence of involuntary movement due to pain was significantly lower in DPNB and combination groups, as compared with tetracaine gel group (30.2% and 26.7% vs 45.3%, *P = *.042 and *P = *.011, respectively) (Figure 3).

## 4 DISCUSSION

This is the first randomized, double‐blind, placebo‐controlled trial to demonstrate that DPNB with or without tetracaine gel instilled reduced overall pain and kept HR and MAP comparatively stable during rigid cystoscopy compared with tetracaine gel, especially when the cystoscope passed through the most painful membranous urethra part. In addition, no adverse events were observed in DPNB with or without tetracaine gel. Our findings suggest that analgesia provided by DPNB with or without topical intraurethral tetracaine gel is superior to that provided by tetracaine gel for male patients undergoing rigid cystoscopy.

Cystoscopy plays an important role in both diagnosis and treatment process in urology. Although flexible cystoscopy is routinely performed in western urology clinics, it is still applied in clinics especially in developing countries for the lower cost, better optical area, and easier to orientate. However, due to the stiffness of rigid cystoscope sheath, discomfort and even pain is common, which derives from the friction between the wall of the urethra or bladder mucosa and the sheath. Our findings demonstrated patients with topical intraurethral administration of local anesthetic tetracaine gel feel more pain during rigid cystoscopy. Even under general anesthesia, men still suffer discomfort and pain during cystoscopy.^3^, ^12^ Thus, an effective analgesia regimen seems to be urgent for men undergoing rigid cystoscopy.

Due to the special nature of the male urethral physiological structure, men are more prone to suffer more pain than women during cystoscopy. Male patients feel the severest pain when the cystoscope passes through the membranous urethra during rigid or flexible cystoscopy. Song et al^9^ did autopsy on males found that membranous urethral was innervated by branches of DNP. In addition, evidence suggests that DNP branches innervate urethra mucosa.^10^ Thus, we proposed DPNB might be effective to reduce pain in men undergoing rigid cystoscopy. Our findings demonstrated that DPNB alone or in combination with tetracaine gel can significantly alleviate pain in the male patients during rigid cystoscopy compared with tetracaine gel alone (both *P* < .0001). In our patients, their VAS scores increased as cystoscope inserted and peaked (median [interquartile]: 5 [4–6]) at the inspection of external sphincter when the tip of cystoscope went through the membranous urethra, indicating most pain results from where the external sphincter is located, among the stages of the cystoscopy. This result also validated why the VAS at cystoscopic inspection of external sphincter was set as the primary outcome of our study. At the primary outcome, DPNB produced a significant reduction in median VAS by 2 units in both DPNB and combination groups, representing a 33% reduction, compared with tetracaine gel group (both *P* < .0001). This reduction can be considered clinically meaningful according to the Initiative on Methods, Measurement, and Pain Assessment in Clinical Trials recommendations and several clinical trials relevant to pain.^13^, ^14^ Additionally, after cystoscope passed through membranous urethra, DPNB could alleviate pain and discomfort for subsequent inspection of prostate and bladder in DPNB and combination groups, resulting in a 2‐point reduction of median VAS, compared with tetracaine gel group (*P* < .001 and *P* < .0001, respectively). And most importantly, DPNB with or without tetracaine gel reduced overall pain for men undergoing rigid cystoscopy compared with tetracaine gel. Furthermore, HR and MAP were significantly lower in the DPNB and combination groups when compared to tetracaine gel group during the procedure, which is consistent with the VAS result. Taken together, our results suggest that DPNB could provide better analgesia than tetracaine gel for men undergoing rigid cystoscopy.

The application of local anesthetic in cystoscopy is still in controversy,^15^, ^16^, ^17^ and most of studies used lidocaine gel as topical intraurethral anesthesia. Tetracaine gel is routinely used in rigid cystoscopy in our hospital for its more effective and longer action time than lidocaine gel.^12^, ^18^ However, we found the VAS score in tetracaine gel group was the highest among the three groups, suggesting that the analgesic effect of tetracaine gel was weak during the procedure in our patients. And the adjunctive administration of tetracaine gel in the patients with DPNB could not reduce pain severity further compared to those with DPNB alone, indicating DPNB may play a key analgesic role in our cystoscopy study.

It has been reported that patients may experience penile erection during cystoscopy and the incidence could be increased with general anesthesia compared to spinal anesthesia.^19^ However in our study, no penile erection occurred during the examination in all the groups. There were some studies reported that DPNB might injure the blood vessels, which subsequently could cause hematoma even local anesthetic intoxication because the local anesthetic goes into blood.^20^ However, in our study, no hematoma, toxicosis and other adverse events were observed in all patients whether with DPNB or not. But, our findings demonstrated that patients with DPNB using ropivacaine had lower incidence of involuntary movement derived from pain, which might be ascribed to the lower pain severities in patients with DPNB, compared with those with tetracaine gel.

Recent studies suggest that DPNB should be done under ultrasound guidance by well‐trained anesthesiologist, which could result in a more accurate nerve blocking and reducing relevant complications.^21^, ^22^ However, it was also reported that there was no significant difference between ultrasound guided DPNBs and anatomical landmark guided DPNBs, and it takes longer time to perform ultrasound guided DPNBs.^23^, ^24^ Therefore, we used anatomical landmark to perform the nerve block.

Our study has limitations. DPNB is an easy and feasible technique, which can be performed by an individual who is trained in the prescribing and administration of local anesthetic.^25^ Although our study showed DPNB alleviated pain in men undergoing rigid cystoscopy compared with intraurethral anesthesia with tetracaine gel, the performance of the block needs trained practitioner and takes extra time (approximately 5 minutes in our study). In addition, our study demonstrated DPNB can relive the pain in men at cystoscopic inspection of the most painful part external sphincter, resulting in a 2‐point drop of VAS. Patients overall appraisal for the satisfaction with their pain management and how willing they would have the same procedure repeated with the same analgesia such parameters would be more clinically relevant to assess clinically important pain reduction as compared with a 2‐point reduction in VAS. Those parameters worth applying in future relevant investigation in assessment of the clinical importance of DPNB for analgesia in men undergoing cystoscopy.

Flexible cystoscopy is routinely performed in western nations. DPNB might be effective for analgesia in men during flexible cystoscopy since our rigid cystoscopy study demonstrated that DPNB could significantly reduce pain and discomfort especially when cystoscope passed through membranous urethra, which is also the most painful moment for flexible cystoscopy. DPNB need further investigation of its analgesia in men during flexible cystoscopy.

## 5 CONCLUSION

DPNB is a safe technique and provides better analgesia for men undergoing diagnostic rigid cystoscopy compared to topical intraurethral anesthesia with tetracaine gel.

## CONFLICT OF INTEREST

None to declare.
